# Excess Glutamate May Cause Dilation of Retinal Blood Vessels in Glutamate/Aspartate Transporter-Deficient Mice

**DOI:** 10.1155/2019/6512195

**Published:** 2019-11-11

**Authors:** Takayuki Gonome, Yuting Xie, Saeko Arai, Kodai Yamauchi, Natsuki Maeda-Monai, Reiko Tanabu, Takashi Kudo, Mitsuru Nakazawa

**Affiliations:** Department of Ophthalmology, Hirosaki University Graduate School of Medicine, Hirosaki, Japan

## Abstract

**Purpose:**

To investigate the longitudinal findings of fundus features and spectral-domain optical coherence tomography (SD-OCT) to characterize the morphologic features in a mouse model of defective glutamate/aspartate transporter (GLAST^−/−^ mice).

**Materials and Methods:**

The fundus findings and SD-OCT images were longitudinally recorded at five time points from postnatal (P) 22 to P156 in GLAST^−/−^ mice. As a control wild type, age-matched C57BL/6J mice were employed. The mouse retina was subdivided into five layers, and the thickness of each layer was longitudinally measured by InSight® using SD-OCT pictures. The SD-OCT findings were compared with the histologic appearances. The diameter of the retinal blood vessels was measured by the ImageJ® software program using SD-OCT images. The data were statistically compared between both age-matched mouse groups.

**Results:**

The retinal blood vessels appeared more dilated in GLAST^−/−^ mice than in wild-type mice. This tendency was statistically significant at all time points after P44 by analyses using SD-OCT images. The ganglion cell complex (GCC) and outer nuclear layer (ONL) were significantly thinner in GLAST^−/−^ mice at all time points after P80 than in the wild-type mice. This tendency was more clearly indicated by SD-OCT than histologic sections.

**Discussion:**

In the present study, we found for the first time the dilation of the retinal blood vessels and the thinning of the ONL in GLAST^−/−^ mice, in addition to the thinning of the GCC.

## 1. Introduction

Glutamate is a major excitatory neurotransmitter of the mammalian retina, and its uptake is essential for neurotransmission at glutamatergic synapses [1]. In the mammalian retina, five subtypes of glutamate transporter are expressed [2]. Among them, glutamate/aspartate transporter (GLAST) is expressed only in Müller cells, and it removes glutamate from the extracellular space [3–6]. This system contributes to glutamate homeostasis in the retina. Therefore, deficient or dysfunction of GLAST leads to elevation of glutamate concentration in the retina.

Previous studies have shown that the glutamate uptake by GLAST into Müller cells provides a substrate for synthesizing glutathione, which is an important radical scavenger composed of a tripeptide of glutamate, cysteine, and glycine. Glutathione has a strong protective role against oxidative stress as an antioxidant in the retina. In addition, the glutamate uptake is a rate-limiting step in glial glutathione synthesis [7, 8]. Therefore, GLAST expressed in Müller cells is essential not only for keeping the extracellular glutamate concentration below the neurotoxic level but also for maintaining the glutathione levels in Müller cells by transporting glutamate into the cells and providing the substrate for glutathione synthesis.

GLAST deficient (GLAST^−/−^) mice are a recognized mouse model of normal tension glaucoma (NTG), as they demonstrate progressive retinal ganglion cell loss and optic nerve degeneration without elevated intraocular pressure and show a glaucomatous pathology, including glutamate neurotoxicity and oxidative stress in the retina [9, 10]. Previous studies regarding NTG model mice have mainly focused on the pathologic changes in the inner retina, consisting of the retinal nerve fiber layer (RNFL) and the retinal ganglion cell layer (GCL) [9, 10]. Although impaired glutathione synthesis causes oxidative stress in the whole retina [11], to our knowledge, no study has described the changes in the retinal layers other than RNFL and GCL. Therefore, in this study, we evaluated the changes in all retinal layers in GLAST^−/−^ mice using spectral-domain optical coherence tomography (SD-OCT). In addition, we investigated the relationship between SD-OCT findings and morphological changes evaluated by histological analyses.

In addition, glutamate is the most common trigger for neurovascular coupling (NVC) in the brain [12]. NVC is the mechanism whereby an increase in neuronal activity leads to local elevation in cerebral blood flow by significant vasodilation of neighboring microvessels to match the metabolic requirements of firing neurons [13]. Among them, astrocytes, in which GLAST is mainly expressed in the cerebellum, play a major role in the communication between activated neurons and blood vessels [14–17]. The function of astrocytes in NVC is reportedly related to their ability to release vasoactive messengers [18], such as epoxyeicosatrienoic acids [19, 20] and potassium [21], through multiple channels, such as large-conductance Ca^2+^-activated K^+^ channels on astrocytes [22] or the inward rectifier K^+^ [23] and TRPV4 channels on microvessels [24]. Furthermore, pericytes, which are contractile cells on capillaries that exist in the retina and brain [25], contribute to vasodilation in the central nervous system. Neuronal activity and glutamate evoke the release of messengers that dilate capillaries by actively relaxing pericytes. Dilation is mediated by prostaglandin E2 but requires nitric oxide release to suppress vasoconstricting 20-hydroxyeicosatetraenoic acid synthesis. Pericytes are major regulators of the cerebral blood flow [26].

We therefore also evaluated whether or not the retinal blood vessels were dilated in GLAST^−/−^ mice using SD-OCT. We suspected that dilation of the retinal blood vessels in GLAST^−/−^ mice might indirectly indicate a high concentration of glutamate in the retina and that glutamate might also contribute to NVC in the retina.

## 2. Materials and Methods

### 2.1. Experimental Animals

All experimental procedures performed in this study conformed to the regulations of the Association for Research in Vision and Ophthalmology (ARVO) Statement for the Use of Animals in Ophthalmic and Vision Research and were approved by the Institutional Committee of Ethics for animal experiments (Approval number: M12023).

GLAST^−/−^ mice were generously provided by Dr. Takayuki Harada (Visual Research Project, Tokyo Metropolitan Institute of Medical Science, Tokyo, Japan) [27]. C57BL/6J mice were purchased from Clea, Japan (Tokyo, Japan), and were used as wild-type controls. The mice were kept in the Hirosaki University Graduate School of Medicine Animal Care Service Facility under a cycle of 12 h of light (50 lx illumination) and 12 h of darkness (<10 lx environmental illumination) in an air-conditioned atmosphere. Mice were given free access to food and water.

### 2.2. SD-OCT Examination and Fundus Photography

**Figure 1:**
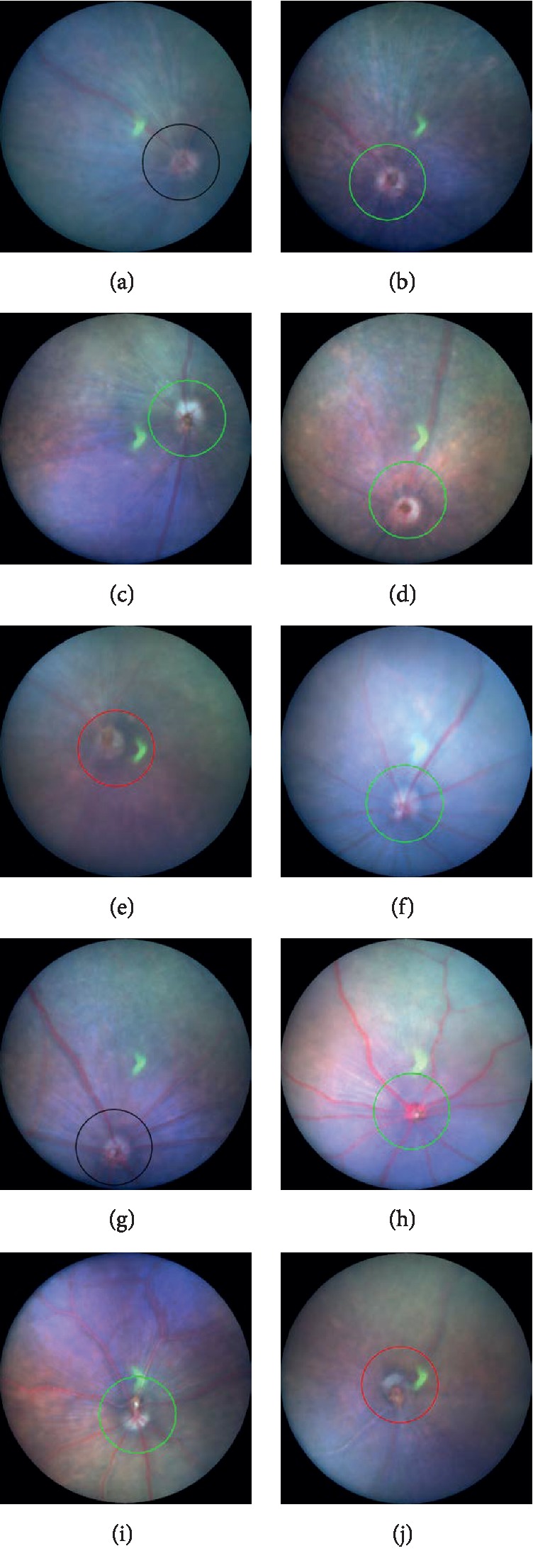
Representative fundus pictures of C57BL/6J (a–e) and GLAST^−/−^ (f–j) mice. (a–e) The fundus findings at postnatal (P) 22, P36, P72, P106, and P148 of C57BL/6J mice, respectively. (f–j) The fundus findings at P22, P44, P80, P114, and P156 of GLAST^−/−^ mice, respectively. Circles indicate the line at which the SD-OCT images were created.

SD-OCT and fundus photography were performed according to the methods previously described using a Micron® IV (Phoenix Research Labs, Pleasanton, CA, USA) [28, 29]. In brief, SD-OCT and fundus photography were carried out at 5 time points from postnatal (P) day 22 to P156 (P22, P44, P80, P114 and P156) for GLAST^−/−^ mice and at 5 time points from P22 to P148 (P22, P36, P72, P106 and P148) for C57BL/6J mice. Four to five mice were examined at a time. The mice were anesthetized with an intraperitoneal injection of a mixture of medetomidine hydrochloride (0.315 mg/kg), midazolam (2.0 mg/kg), and butorphanol tartrate (2.5 mg/kg). The pupils were dilated with the instillation of eye drops containing a mixture of 0.5% tropicamide and 0.5% phenylephrine hydrochloride. The mouse ocular fundus was simultaneously monitored by a fundus camera, and the position of the retinal SD-OCT image was set circumferentially around the optic disc by considering potential structural differences between the upper and lower hemispheres of the mouse eyes (360°; diameter, 500 *μ*m; 140 *μ*m away from the optic disc margin; Figure 1) [30]. The corneal surface was protected using a 1.5% hydroxyethyl cellulose solution. Fifty images were averaged to eliminate the projection artifacts. The quantitative analysis of the acquired OCT images was performed using the InSight® software program (Phoenix Research Labs). During all experimental procedures, the physical condition of the mice was frequently monitored by inspection and gentle palpation by the researchers.

### 2.3. The Analyses of the Retinal Layer Thickness

We measured the thickness of the retina by dividing it into five layers. From inside, the first layer consisted of the RNFL, GCL, and inner plexiform layer (IPL); this layer is clinically regarded as the ganglion cell complex (GCC). The second layer consisted of the inner nuclear layer (INL) and the outer plexiform layer (OPL); the third layer consisted of the outer nuclear layer (ONL). The fourth layer consisted of the photoreceptor inner segment (IS) and outer segment (OS) layers. The deepest layer consisted of the retinal pigment epithelium (RPE) and choroid (S1).

Segmentation was performed using the InSight® software program (Phoenix Research Labs), as previously reported [28–30]. The borderlines between each sublayer were automatically identified by the software program using the SD-OCT images and were manually corrected by the researchers when necessary. The average distance (*μ*m) between each borderline was calculated using the raw data summarized in the Excel® file generated by the InSight® software program. The data obtained from both eyes of the same animal were averaged. The overall average retinal layer thickness was presented as the mean ± standard deviation.

### 2.4. Measurement of Diameter of the Retinal Vessels Using SD-OCT

The diameter of the retinal vessels irrespective of arteries and veins was indirectly measured using SD-OCT images. Because the retinal blood vessels have sharply demarcated shadows in the SD-OCT picture and the width of these shadows exactly corresponds to the vessel diameter, we measured the width of the vessel shadows using ImageJ® software program and expressed the value in pixels. We measured the width of all vessel shadows, regardless of whether they were arteries or veins, which appeared in each of the SD-OCT images and expressed them as the median ± quartile deviation. If the vessel shadow was not clear enough to be identified, then, it was excluded from the measurement.

### 2.5. Histologic Examinations

Histologic examinations were performed using eyes enucleated from GLAST^−/−^ mice on P67 and P128 and C57BL/6J mice on P66 and P120. Immediately after euthanasia by luxation of the cervical spine, the eyes were excised under a microscope. To prevent the possibility of artificial retinal detachment during further processing, an aliquot of 2% glutaraldehyde and 2% paraformaldehyde solution (pH 7.4) was injected into the anterior chamber or vitreous chamber through the corneal limbus. After fixation in the same solution for 2 h at room temperature, the eyeballs were refixed in 4% paraformaldehyde solution at pH 7.0 for 24 h at 4°C. Paraffin embedding, sectioning, and hematoxylin and eosin (HE) staining were performed as previously described [28, 30]. The HE-stained sections were photographed under a light microscope (DP-71; Olympus, Tokyo, Japan). The histological findings were compared to the corresponding findings from SD-OCT images.

### 2.6. Statistical Analyses

The statistical analyses of the data obtained in the present study were performed using the SPSS software program (version 22, Statistical Package for the Social Sciences, Chicago, IL, USA). The segmentation data from the two groups were compared using a two-way repeated analysis of variance (two-way repeated ANOVA) after the normality of each distribution was confirmed by the Shapiro–Wilk test. The parametric or nonparametric method was chosen depending on the presence of normality. Student's *t*-test was performed to analyze differences in SD-OCT segmentation between similar age groups (GLAST^−/−^ vs. C57BL/6J mice: P22 vs. P22, P44 vs. P36, P80 vs. P72, P114 vs. P106, and P156 vs. P148, respectively). The similar age groups include pairs of GLAST^−/−^ and C57BL/6J mice of birth dates within 10-day difference. The Mann–Whitney *U*-test was used to compare the diameter of the retinal blood vessels between GLAST^−/−^ and C57BL/6J mice. *P* values of <0.05 were considered to indicate statistical significance.

## 3. Results

### 3.1. Fundus Findings of C57BL/6J and GLAST^ −/−^ Mice

**Figure 2:**
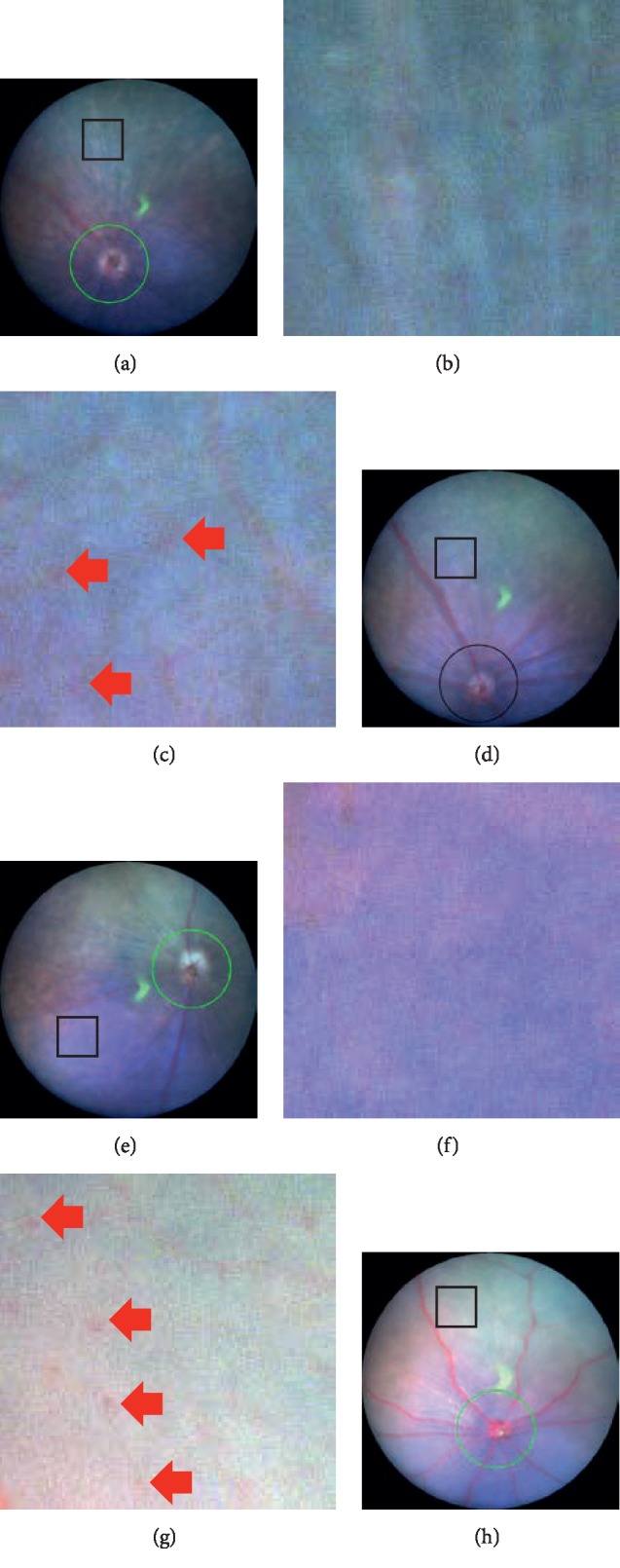
Magnified fundus pictures of C57BL/6J and GLAST^−/-^ mice. The rectangular area in each panel (a, d, e, and h) is magnified in the inset (b, c, f, and g, respectively). Panels a and c correspond to the fundus findings at P36 and P72 of C57BL/6J mice, respectively. Panels b and d correspond to the fundus findings at P44 and P80 of GLAST^−/-^ mice, respectively. The square represents 250  *μ*m × 250 *μ*m in panels (a) through (d). All insets are the same magnifications. Arrows indicate capillaries.

The long-term changes in the fundus findings of both C57BL/6J and GLAST^−/−^ mice are presented in Figure 1. In contrast to the findings in the C57BL/6J mice (Figures 1(a)–1(e)), the retinal blood vessels appeared qualitatively more dilated and pinkish in the GLAST^−/−^ mice from P22 to P114 (Figures 1(f)–1(j)). Furthermore, not only the large retinal blood vessels but also retinal capillaries appeared more dilated in GLAST^−/−^ mice than in C57BL/6J mice (Figure 2). There have been no previous reports describing the characteristics of the fundus findings of GLAST^−/−^ mice. We therefore report for the first time the fundus changes of GLAST^−/−^ mice.

### 3.2. Qualitative and Quantitative Analyses of the Retinal Layer Obtained by SD-OCT

**Figure 3:**
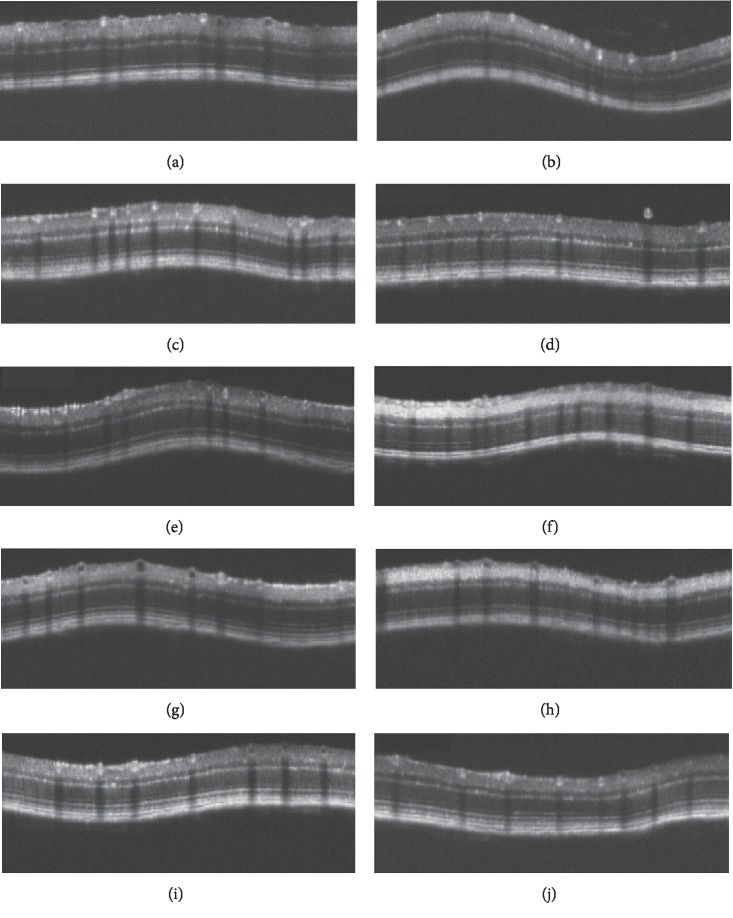
Representative SD-OCT images of C57BL/6J (a–e) and GLAST^−/−^ (f–j) mice. (a–e) The fundus findings at P22, P36, P72, P106, and P148 of C57BL/6J mice, respectively. (f–j) The fundus findings at P22, P44, P80, P114, and P156 of GLAST^−/−^ mice, respectively.

We analyzed the SD-OCT images of both C57BL/6J and GLAST^−/−^ mice in order to qualitatively characterize the SD-OCT findings (Figure 3). Typical SD-OCT findings in C57BL/6J mice obtained from P22 to P148 are shown in Figures 3(a)–3(e). The SD-OCT findings in GLAST^−/-^ mice obtained from P22 to P156 are shown in Figures 3(f)–3(j). There were no marked differences in the reflectivity of each retinal layer between the two mouse groups throughout the observation periods.

**Figure 4:**
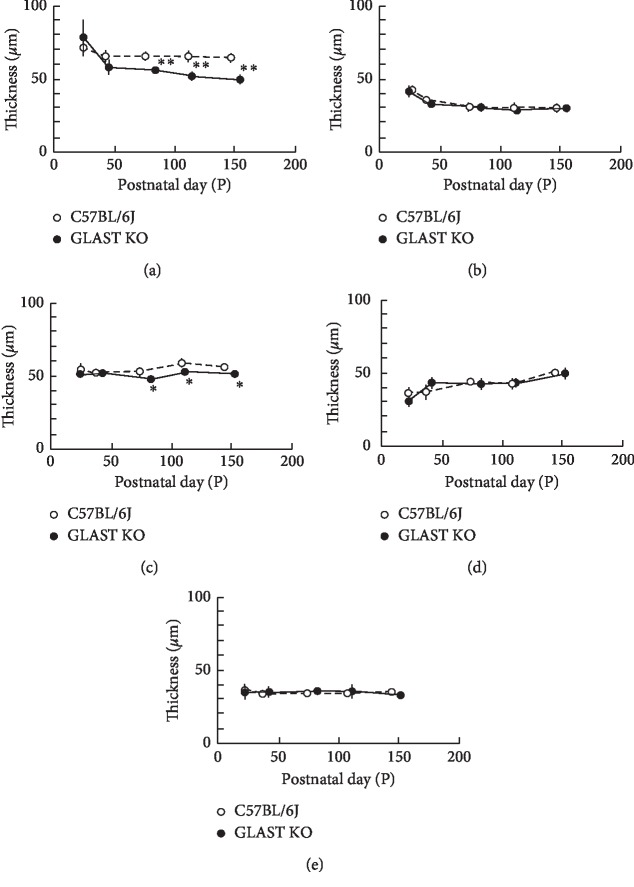
The longitudinal changes in the thickness of the retinal layers. Open circles, C57BL/6J; closed circles, GLAST^−/−^ mice. (a) Thickness changes in the combined retinal nerve fiber, ganglion cell and inner plexiform layers. (b) Thickness changes in the combined inner nuclear and outer plexiform layers. (c) Thickness changes in the outer nuclear layer. (d) Thickness changes in the combined inner segment and outer segment layers. (e) Thickness changes in the combined the retinal pigment epithelium and choroid. Animal numbers: GLAST^−/−^, P22 (*n* = 6), P44∼P114 (*n* = 4), P156 (*n* = 5); C57BL/6J, P22∼P148 (*n* = 4). Statistical significance: ^*∗*^*P* < 0.05; ^*∗∗*^*P* < 0.01 (Student's *t*-test).

**Figure 5:**
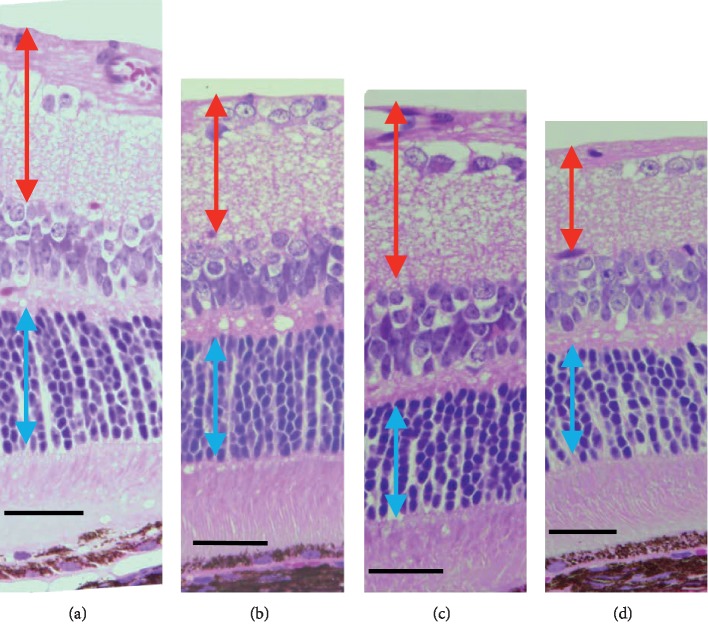
Light microscopic findings by hematoxylin and eosin staining of C57BL/6J (a and c) and GLAST^−/−^ (b and d) mice. (a, c) The C57BL/6J at P66 and P120, respectively. (b, d) The GLAST^−/−^ at P67 and P128, respectively. Red arrows indicate the ganglion cell complex. Blue arrows indicate the outer nuclear layer. Bars indicate 50 *μ*m.

It was previously shown that the GCC was thinner in GLAST^−/−^ mice than in wild-type mice due to the loss of RGCs and thinning of the RNFL [9]. To confirm this observation, we longitudinally and quantitatively analyzed the SD-OCT features in both groups. The results obtained are shown in Figure 4. There were no statistically significant differences in the thickness of the sublayers of INL + OPL, IS/OS, or RPE + choroid layers at any time point between GLAST^−/−^ and C57BL6J mice. However, the thickness of the retinal layers (GCC and ONL) in the GLAST^−/−^ mice was significantly thinner than in the C57BL/6J mice at all time points after P80. The tendency toward thinning of the GCC corresponded with the results of histologic analyses, although the thinning in the ONL was not obvious in the histologic sections (Figure 5).

### 3.3. Quantitative Analyses of the Retinal Blood Vessels Diameter

**Figure 6:**
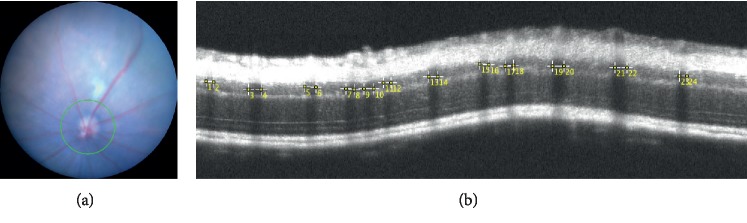
Representative fundus picture (a) and SD-OCT image (b) indicating the method of measuring the retinal vessel diameter using the ImageJ® software program.

**Figure 7:**
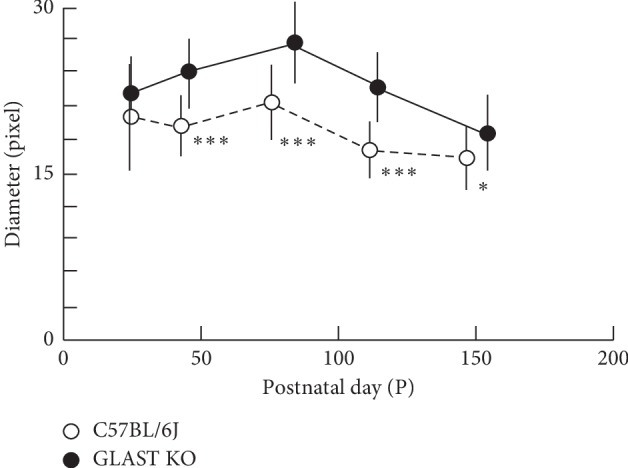
The longitudinal changes in the median of the retinal vessel diameter. Open circles, C57BL/6J; closed circles, GLAST^−/−^ mice. Animal numbers: GLAST^−/−^, P22 (*n* = 6), P44∼P114 (*n* = 4), P156 (*n* = 5); C57BL/6J, P22∼P148 (*n* = 4). Statistical significance: ^*∗*^*P* < 0.05; ^*∗∗∗*^*P* < 0.001 (Mann–Whitney *U*-test).

To confirm whether the retinal blood vessels are more dilated in GLAST^−/−^ mice than in C57BL/6J mice as shown in Figure 1, we indirectly measured the diameter of the retinal blood vessels including both arteries and veins using SD-OCT. Because the vessel shadows appeared to correspond to the diameter of the blood vessels, we measured the width of the vessel shadow using the ImageJ® software program. A typical SD-OCT picture and measurement image by ImageJ® is presented in Figure 6. The longitudinal changes in the median of the diameter in the retinal blood vessels are shown in Figure 7. As expected, the median of the diameter in the retinal blood vessels was wider in GLAST^−/−^ mice than in C57BL/6J mice. There were statistically significant differences between the two groups at all time points after P44 (Figure 7), indicating that the retinal blood vessels of GLAST^−/−^ mice are more dilated than those of wild-type mice. As shown in Figure 7, the retinal blood vessel diameter became largest at P80, and then gradually decreased afterword. The degree of change in the median of the diameter was 30.0% from P80 to P156 in GLAST^−/−^ mice, whereas that in control mice was 21.9% decrease during the same period.

## 4. Discussion

In this study, we first showed the dilation of the retinal blood vessels and thinning of the ONL in GLAST^−/−^ mice, in addition to the thinning of the GCC that has previously been reported [9, 27, 31]. Many reports have been published concerning GLAST^−/−^ mice since Watase et al. first reported GLAST mutant mice in 1998 [32]. In the retina, GLAST is expressed in Müller cells and maintains glutamate homeostasis by taking up extracellular glutamate released for glutamatergic neurotransmission in the retina [1]. In GLAST^−/−^ mice, both excitotoxicity and oxidative stress may lead to RGC degeneration. GLAST is essential not only for keeping the extracellular concentration of glutamate below the neurotoxic level but also for maintaining the glutathione levels in Müller cells by transporting glutamate into the cells. Glutamate functions as a substrate for glutathione synthesis. Because glutathione has a central role in protecting retina against oxidative stress, the glutamate uptake is a rate-limiting step in glial glutathione synthesis [7, 8]. As retinal glutathione is only produced in Müller cells, the impaired glutathione synthesis in Müller cells may cause oxidative stress in the retina.

GLAST^−/−^ mice are a recognized model of NTG as they show the same morphological changes as NTG due to RGC loss [9, 10]. It was recently reported that the free-radical scavenger edaravone and the mucolytic agent N-acetylcystein suppress RGC loss in GLAST^−/−^ mice [27, 31]. There have been many other interventional studies exploring the suppression of RGC loss in GLAST^−/−^ mice as well [33–35].

In the present study, we confirmed the previously reported characteristic thinning of GCC using SD-OCT. At all time points after P80, significant thinning of GCC was detected on SD-OCT findings (Figure 4). Because it was hard to discriminate the RGC from RNFL by SD-OCT, we measured the GCC layer including the IPL as the superficial layer of the retina. In this regard, therefore, the SD-OCT measurement is not very sensitive as a histologic analysis. Although this may be a limitation of the SD-OCT method, this modality has an advantage over histologic analyses because it is repeatable and less invasive.

The ONL was significantly thinner in GLAST^−/−^ mice than in the wild-type mice after P80 in our study suggesting that the photoreceptors were also damaged to some extent, at least after P80. Because neither cone nor rod photoreceptors express glutamate receptors, it is unlikely that cell death was induced by the glutamate-induced excitotoxicity that may happen in the RGC. However, although the exact mechanisms are largely unknown, it is still possible that the thinning of the ONL may have resulted from photoreceptor cell damage due to oxidative stress caused by the decrease in glutathione in the retina.

Interestingly, we found that the diameter of the retinal blood vessels was significantly larger in GLAST^−/−^ mice than in wild-type mice and not only the large retinal vessels but also the retinal capillaries were dilated in GLAST^−/−^ mice compared to C57BL/6J mice. This result supports previous reports suggesting that glutamate plays a major role in NVC and vasodilation [12, 26]. Our results suggest that excess glutamate may cause dilation of the blood vessels in the retina.

Of note, GLAST^−/−^ mice are also recognized as an animal model for schizophrenia, as they exhibit behavioral abnormalities simulating the positive symptoms of schizophrenia [36–38]. Schizophrenia is a serious mental disorder that affects up to 1% of the general population worldwide; however, the exact neurochemical mechanisms involved remain unknown [39]. Regarding the etiology, although various hypotheses have been proposed, the glutamate theory is one of the main hypotheses that have been offered [39, 40]. The glutamate hypothesis is based on the finding that antagonists of the N-methyl-d-aspartate (NMDA) receptors induce schizophrenia-like symptoms in healthy individuals and exacerbate symptoms in patients with schizophrenia [40]. In addition, many reports have shown that schizophrenia indicates abnormalities of the blood flow in the brain [41–44]. For example, Talati et al. noted an increased hippocampal cerebral blood volume but a normal cerebral blood flow in patients with schizophrenia [41]. In addition, Ku et al. found the impaired maintenance of a constant cerebral blood flow and a delayed cerebrovascular autoregulatory response in patients with schizophrenia [43]. These findings may suggest that schizophrenia represents the impaired autoregulation of the necessary blood supply to required sites for physiological brain activity. In the brain, GLAST and glutamate transporter-1 are the major transporters that take up synaptic glutamate in order to maintain optimal extracellular glutamate levels, thereby preventing both the accumulation of glutamate in the synaptic cleft and ensuing excitotoxicity [36]. GLAST is expressed in astrocytes in the cerebellum and the forebrain regions, including the cerebral cortex and hippocampus [38]. A genetic abnormality of glutamate transporter was previously found in patients with schizophrenia [45, 46]. In addition, the GLAST expression is decreased in patients with schizophrenia as compared to healthy subjects according to a postmortem schizophrenic brain analysis [47]. Considering these previous results regarding the relationship between an impaired blood flow and glutamate in schizophrenia, retinal vasodilation in GLAST^−/−^ mice may be somehow related to the abnormal extracellular concentration of glutamate in this animal model.

Several limitations associated with the present study warrant mention. First, although we speculate the photoreceptor damage may have been induced by oxidative stress in GLAST^−/−^ mice, we did not measure the levels of glutathione and oxidative stress in the retina quantitatively. Further studies will be needed in order to clarify whether or not oxidative stress is actually increasing in the retina of GLAST^−/−^ mice. Second, the diameter of the retinal blood vessels may have been genetically determined to be large due to the mechanisms other than the high concentration of glutamate in GLAST^−/−^ mice. Therefore, whether or not an impaired glutamate uptake really causes vasodilation in the retina of GLAST^−/−^ mice should be clarified. Third, it is not clear whether the sharply demarcated shadows appeared in SD-OCT correspond to the inner or outer diameters of the vessels. Because recently advanced OCT angiography technique detects intravascular red blood cells, it is probable that the vessel shadows correspond to the inner diameters of the vessels. However, this point needs to be further clarified by studies including functional evaluation, such as flow measurement. Forth, we also need to clarify whether the retinal vascular dilation is associated with oxidative stress. It has been reported that cigarette smoking may cause retinal vein dilation [48]. Because cigarette smoking is considered to increase the risk of cardiovascular diseases via oxidative stress [49, 50], it is not contradictory to speculate that oxidative stress is related to retinal vascular dilation in some conditions.

In conclusion, we observed the dilation of the retinal blood vessels and thinning of the ONL, in addition to the thinning of the GCC in GLAST^−/−^ mice. We hypothesize that the impaired uptake of extracellular glutamate into Müller calls may cause dilation of the retinal blood vessels and damage to the GCC and photoreceptors in GLAST^−/−^ mice. Further studies are needed to clarify these hypotheses.

## Data Availability

The minimal data set used to support the findings of this study is included with the supplementary information file.

## Supplementary Materials

Supplementary Materials The definition of retinal and RPE/choroid layers on SD-OCT and a comparison between a representative SD-OCT image and histologic findings.

## References

[1] Rauen T., Wiessner M. (2000). Fine tuning of glutamate uptake and degradation in glial cells: common transcriptional regulation of GLAST1 and GS. *Neurochemistry International*.

[2] Pow D. V. (2001). Amino acids and their transporters in the retina. *Neurochemistry International*.

[3] Rauen T., Taylor W. R., Kuhlbrodt K., Wiessner M. (1997). High-affinity glutamate transporters in the rat retina: a major role of the glial glutamate transporter GLAST-1 in transmitter clearance. *Cell and Tissue Research*.

[4] Derouiche A., Rauen T. (1995). Coincidence of L-glutamate/L-aspartate transporter (GLAST) and glutamine synthetase (GS) immunoreactions in retinal glia: evidence for coupling of GLAST and GS in transmitter clearance. *Journal of Neuroscience Research*.

[5] Rauen T., Rothstein J. D., Wässle H. (1996). Differential expression of three glutamate transporter subtypes in the rat retina. *Cell and Tissue Research*.

[6] Park C. K., Cha J., Park S. C. (2009). Differential expression of two glutamate transporters, GLAST and GLT-1, in an experimental rat model of glaucoma. *Experimental Brain Research*.

[7] Schulz J. B., Lindenau J., Seyfried J., Dichgans J. (2000). Glutathione, oxidative stress and neurodegeneration. *European Journal of Biochemistry*.

[8] Huster D., Reichenbach A., Reichelt W. (2000). The glutathione content of retinal Müller (glial) cells: effect of pathological conditions. *Neurochemistry International*.

[9] Harada T., Harada C., Nakamura K. (2007). The potential role of glutamate transporters in the pathogenesis of normal tension glaucoma. *Journal of Clinical Investigation*.

[10] Kimura A., Guo X., Noro T. (2014). Valproic acid prevents retinal degeneration in a murine model of normal tension glaucoma. *Neuroscience Letters*.

[11] Tanito M., Nishiyama A., Tanaka T. (2002). Change of redox status and modulation by thiol replenishment in retinal photooxidative damage. *Investigative Ophthalmology & Visual Science*.

[12] Zuccolo E., Kheder D. A., Lim D. (2019). Glutamate triggers intracellular Ca^2+^ oscillations and nitric oxide release by inducing NAADP- and InsP_3_ -dependent Ca^2+^ release in mouse brain endothelial cells. *Journal of Cellular Physiology*.

[13] Guerra G., Lucariello A., Perna A., Botta L., De Luca A., Moccia F. (2018). The role of endothelial Ca^2+^ signaling in neurovascular coupling: a view from the lumen. *International Journal of Molecular Sciences*.

[14] Zonta M., Angulo M. C., Gobbo S. (2003). Neuron-to-astrocyte signaling is central to the dynamic control of brain microcirculation. *Nature Neuroscience*.

[15] Iadecola C., Nedergaard M. (2007). Glial regulation of the cerebral microvasculature. *Nature Neuroscience*.

[16] Koehler R. C., Roman R. J., Harder D. R. (2009). Astrocytes and the regulation of cerebral blood flow. *Trends in Neurosciences*.

[17] Cauli B., Hamel E. (2018). Brain perfusion and astrocytes. *Trends in Neurosciences*.

[18] Lecrux C., Bourourou M., Hamel E. (2019). How reliable is cerebral blood flow to map changes in neuronal activity?. *Autonomic Neuroscience*.

[19] Gebremedhin D., Ma Y. H., Falck J. R., Roman R. J., VanRollins M. (1992). Mechanism of action of cerebral epoxyeicosatrienoic acids on cerebral arterial smooth muscle. *American Journal of Physiology-Heart and Circulatory Physiology*.

[20] Peng X., Carhuapoma J. R., Bhardwaj A. (2002). Suppression of cortical functional hyperemia to vibrissal stimulation in the rat by epoxygenase inhibitors. *American Journal of Physiology-Heart and Circulatory Physiology*.

[21] Filosa J. A., Bonev A. D., Straub S. V. (2006). Local potassium signaling couples neuronal activity to vasodilation in the brain. *Nature Neuroscience*.

[22] Girouard H., Bonev A. D., Hannah R. M., Meredith A., Aldrich R. W., Nelson M. T. (2010). Astrocytic endfoot Ca^2+^ and BK channels determine both arteriolar dilation and constriction. *Proceedings of the National Academy of Sciences*.

[23] Longden T. A., Nelson M. T. (2015). Vascular inward rectifier K^+^channels as external K^+^sensors in the control of cerebral blood flow. *Microcirculation*.

[24] Dunn K. M., Hill-Eubanks D. C., Liedtke W. B., Nelson M. T. (2013). TRPV4 channels stimulate Ca^2+^-induced Ca^2+^ release in astrocytic endfeet and amplify neurovascular coupling responses. *Proceedings of the National Academy of Sciences*.

[25] Mishra A., O’Farrell F. M., Reynell C., Hamilton N. B., Hall C. N., Attwell D. (2014). Imaging pericytes and capillary diameter in brain slices and isolated retinae. *Nature Protocols*.

[26] Hall C. N., Reynell C., Gesslein B. (2014). Capillary pericytes regulate cerebral blood flow in health and disease. *Nature*.

[27] Sano H., Namekata K., Kimura A. (2019). Differential effects of N-acetylcysteine on retinal degeneration in two mouse models of normal tension glaucoma. *Cell Death and Disease*.

[28] Adachi K., Takahashi S., Yamauchi K., Mounai N., Tanabu R., Nakazawa M. (2016). Optical coherence tomography of retinal degeneration in Royal College of surgeons Rats and its correlation with morphology and electroretinography. *PLoS One*.

[29] Monai N., Yamauchi K., Tanabu R., Gonome T., Ishiguro S., Nakazawa M. (2018). Characterization of photoreceptor degeneration in the rhodopsin P23H transgenic rat line 2 using optical coherence tomography. *PLoS One*.

[30] Tanabu R., Sato K., Monai N. (2019). The findings of optical coherence tomography of retinal degeneration in relation to the morphological and electroretinographic features in RPE65^−/−^ mice. *PLoS One*.

[31] Akaiwa K., Namekata K., Azuchi Y. (2017). Edaravone suppresses retinal ganglion cell death in a mouse model of normal tension glaucoma. *Cell Death & Disease*.

[32] Watase K., Hashimoto K., Kano M. (1998). Motor discoordination and increased susceptibility to cerebellar injury in GLAST mutant mice. *European Journal of Neuroscience*.

[33] Dong Z., Shinmei Y., Dong Y. (2016). Effect of geranylgeranylacetone on the protection of retinal ganglion cells in a mouse model of normal tension glaucoma. *Heliyon*.

[34] Guo X., Kimura A., Azuchi Y. (2016). Caloric restriction promotes cell survival in a mouse model of normal tension glaucoma. *Scientific Reports*.

[35] Bai N., Hayashi H., Aida T. (2013). Dock3 interaction with a glutamate-receptor NR2D subunit protects neurons from excitotoxicity. *Molecular Brain*.

[36] Pajarillo E., Rizor A., Lee J., Aschner M., Lee E. (2019). The role of astrocytic glutamate transporters GLT-1 and GLAST in neurological disorders: potential targets for neurotherapeutics. *Neuropharmacology*.

[37] Parkin G. M., Udawela M., Gibbons A., Dean B. (2018). Glutamate transporters, EAAT1 and EAAT2, are potentially important in the pathophysiology and treatment of schizophrenia and affective disorders. *World Journal of Psychiatry*.

[38] Karlsson R.-M., Tanaka K., Saksida L. M., Bussey T. J., Heilig M., Holmes A. (2009). Assessment of glutamate transporter GLAST (EAAT1)-deficient mice for phenotypes relevant to the negative and executive/cognitive symptoms of schizophrenia. *Neuropsychopharmacology*.

[39] Javitt D. C. (2010). Glutamatergic theories of schizophrenia. *Israel Journal of Psychiatry and Related Sciences*.

[40] Hu W., MacDonald M. L., Elswick D. E., Sweet R. A. (2015). The glutamate hypothesis of schizophrenia: evidence from human brain tissue studies. *Annals of the New York Academy of Sciences*.

[41] Talati P., Rane S., Skinner J., Gore J., Heckers S. (2015). Increased hippocampal blood volume and normal blood flow in schizophrenia. *Psychiatry Research: Neuroimaging*.

[42] Koike S., Takizawa R., Nishimura Y., Kinou M., Kawasaki S., Kasai K. (2013). Reduced but broader prefrontal activity in patients with schizophrenia during n-back working memory tasks: a multi-channel near-infrared spectroscopy study. *Journal of Psychiatric Research*.

[43] Ku H.-L., Wang J.-K., Lee H.-C. (2017). Cerebral blood flow autoregulation is impaired in schizophrenia: a pilot study. *Schizophrenia Research*.

[44] Kawakami K., Wake R., Miyaoka T., Furuya M., Liaury K., Horiguchi J. (2014). The effects of aging on changes in regional cerebral blood flow in schizophrenia. *Neuropsychobiology*.

[45] Need A. C., McEvoy J. P., Gennarelli M. (2012). Exome sequencing followed by large-scale genotyping suggests a limited role for moderately rare risk factors of strong effect in schizophrenia. *The American Journal of Human Genetics*.

[46] Walsh T., McClellan J. M., McCarthy S. E. (2008). Rare structural variants disrupt multiple genes in neurodevelopmental pathways in schizophrenia. *Science*.

[47] Shan D., Lucas E. K., Drummond J. B., Haroutunian V., Meador-Woodruff J. H., McCullumsmith R. E. (2013). Abnormal expression of glutamate transporters in temporal lobe areas in elderly patients with schizophrenia. *Schizophrenia Research*.

[48] Yanagi M., Misumi M., Kawasaki R. (2014). Is the association between smoking and the retinal venular diameter reversible following smoking cessation?. *Investigative Opthalmology & Visual Science*.

[49] Esen A. M., Barutcu I., Acar M. (2004). Effect of smoking on endothelial function and wall thickness of brachial artery. *Circulation Journal*.

[50] Holay M. P., Paunilar N. P., Joshi P. P., Sahasrabhojney V. S., Tankhiwale S. R. (2004). Effect of passive smoking on endothelial function in healthy adults. *The Journal of the Association of Physicians in India*.

