# Supplementary figures and images for: Excess Glutamate May Cause Dilation of Retinal Blood Vessels in Glutamate/Aspartate Transporter-Deficient Mice
Source: Biomed Res Int. 2019 Nov 11;2019:6512195. doi: 10.1155/2019/6512195 (PMC6881573; doi:10.1155/2019/6512195)

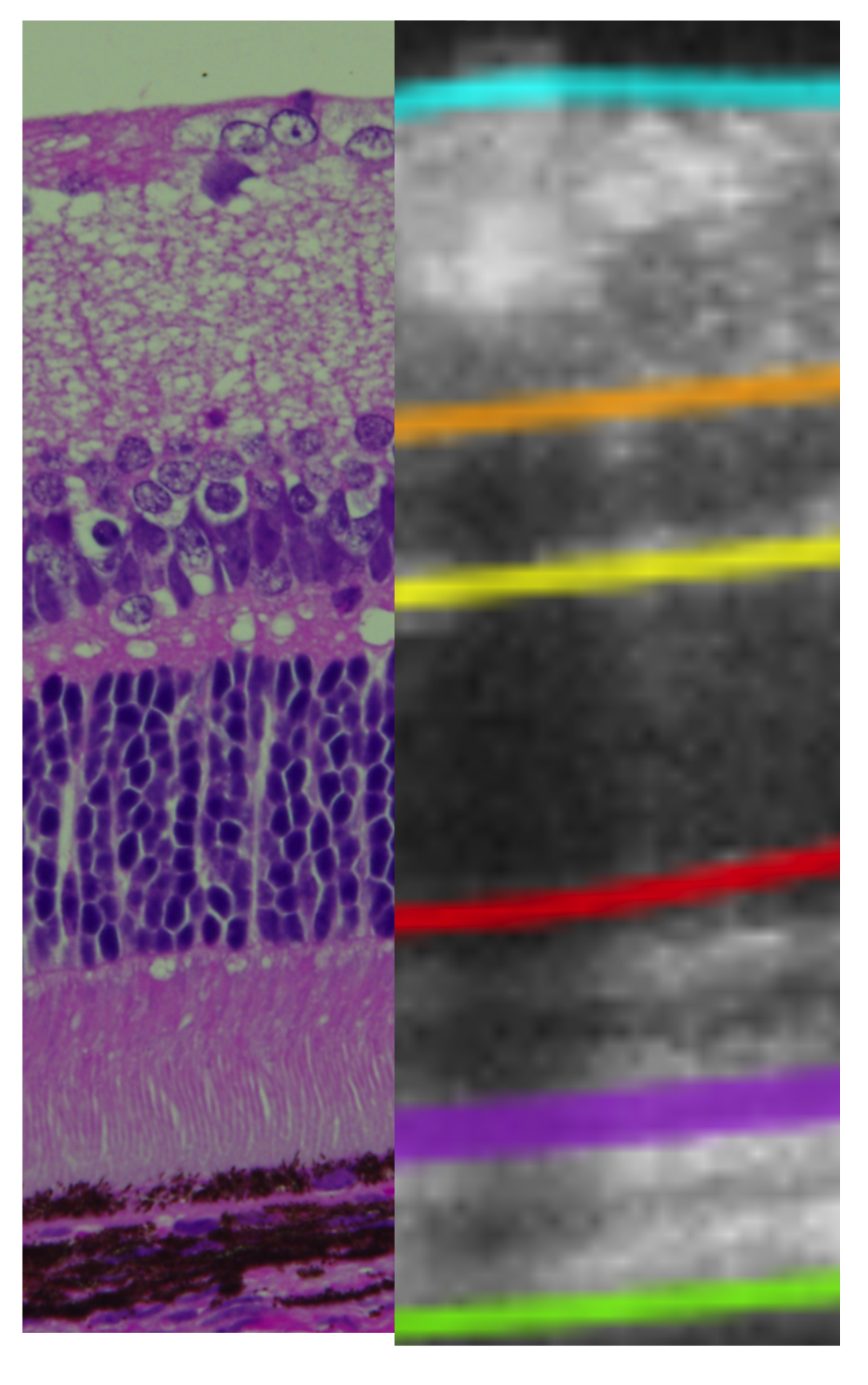

Supplement: Supplementary Materials — The definition of retinal and RPE/choroid layers on SD-OCT and a comparison between a representative SD-OCT image and histologic findings. [file 6512195.f1.jpg]
